# Prognostic impact of Borrmann classification on advanced gastric cancer: a retrospective cohort from a single institution in western China

**DOI:** 10.1186/s12957-020-01987-5

**Published:** 2020-08-13

**Authors:** Xiao-Hai Song, Wei-Han Zhang, Kai-Liu, Xiao-Long Chen, Lin-Yong Zhao, Xin-Zu Chen, Kun-Yang, Zong-Guang Zhou, Jian-Kun Hu

**Affiliations:** 1 Department of Gastrointestinal Surgery and Laboratory of Gastric Cancer, State Key Laboratory of Biotherapy, West China Hospital, Sichuan University and Collaborative Innovation Center for Biotherapy, No. 37 Guo Xue Xiang Street, Chengdu, 610041 Sichuan China; 2 Department of Gastrointestinal Surgery and Laboratory of Digestive Surgery, Institute of Digestive Surgery and State Key Laboratory of Biotherapy and Cancer Center, West China Hospital, Sichuan University and Collaborative Innovation Center for Biotherapy, No. 37 Guo Xue Xiang Street, Chengdu, 610041 Sichuan China

**Keywords:** Gastric cancer, Borrmann type, Clinicopathological features, Prognosis

## Abstract

**Background:**

Due to the controversy over the prognostic significance of Borrmann type in patients with gastric cancer (GC), the present study was to investigate the clinical value of Borrmann type in advanced GC.

**Methods:**

We retrospectively evaluated 2092 patients with advanced GC and subsequently examined the clinicopathological characteristics and prognosis of patients stratified by Borrmann type.

**Results:**

Patients were divided into three groups according to Borrmann type (Borrmann types I+II, III, and IV). Patients with Borrmann types III and IV had larger size, more poorly differentiated tumor type, more advanced tumor stage, and higher chance of involving the entire stomach. The overall survival (OS) rates were significantly different among the three groups (*p* < 0.001). Stratification analysis revealed significant OS rates among the three groups in tumor-node-metastasis (TNM) stage III (*p* < 0.001) and TNM stage IV (*p* = 0.008). Multivariate analysis revealed that Borrmann types, adjuvant chemotherapy, curative resection, and TNM stage were all independent predictors of OS among GC patients. The subgroup analysis indicated that Borrmann type was an independent predictor of OS among GC patients who undergone curative resection and with TNM stage III cancer. However, curative resection and postoperative chemotherapy failed to prolong the survival of patients with Borrmann type IV.

**Conclusions:**

The clinicopathological characteristics and prognosis of patients with three Borrmann types of GC were different. Borrmann type can be simply used as a valuable factor to predict survival in advanced GC patients, especially in those TNM stage III undergoing curative resection. Additionally, more attention should be paid to the treatment for Borrmann type IV GC.

## Introduction

The morbidity of gastric cancer (GC) has decreased over the past decades, but it remains the second leading cause of cancer-associated mortality in China [1]. The survival outcomes of patients with advanced GC are still unsatisfactory in spite of optimized surgery and chemoradiotherapy [2–4]. Therefore, the identification of prognostic factors is necessary, which further renders the establishment of appropriate therapeutic strategies for patients with advanced GC. At present, several clinicopathological parameters, including pathological classification, tumor size, lymph node involvement, and depth of invasion, have been evaluated, aiming at the identification of prognostic indicators influencing GC patients [5–8]. Notably, tumor invasion depth and lymph node involvement are generally considered as the most vital prognostic indicators in GC [8].

The Borrmann classification system, first proposed in 1926, has been prevalently adopted for the description of the gross or endoscopic findings easily based on macroscopic pathological assessment or endoscopy after resection [9, 10]. The advanced GC can be divided into four types based on macroscopic findings (Borrmann types I to IV). Despite certain investigations on the clinicopathological features of Borrmann type IV GC [9–11], its prognostic significance on GC patients remains unclear [12]. To this end, the present study was designed to elucidate the clinical value of Borrmann type in advanced GC by comparing the clinicopathological characteristics and prognosis in GC patients with different Borrmann types.

## Patients and methods

### Patients

**Fig. 1:**
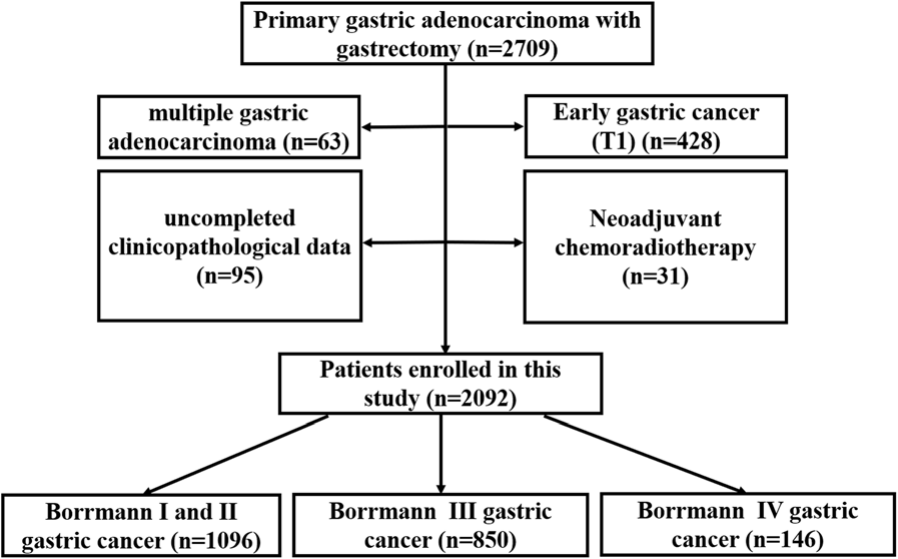
The flow chart of included patients in this study

From January 2009 to December 2015, a total of 2709 consecutive gastric cancer patients who underwent gastrectomy at the Department of Gastrointestinal Surgery of West China Hospital, Sichuan University, were enrolled in this retrospective study. Patients were included based on the following criteria: (1) patients with primary advanced GC confirmed by pathological examination and (2) patients with complete medical data. Patients would be excluded based on the following conditions: (1) patients with early-stage GC (T1; *n* = 428), (2) patients undergoing neoadjuvant chemoradiotherapy (*n* = 31), (3) incomplete medical records (*n* = 95), and (4) multiple gastric adenocarcinoma (*n* = 63). According to the exclusion criteria, 2092 eligible patients retained and were further assessed (Fig. 1). Firstly, patients in this study were divided into four groups according to the Borrmann type (I, II, III, and IV). We found similar biological behaviors between Borrmann type I and type II GC. In consideration of the small sample of type I GC patients, patients with types I and II were combined. As a result, patients enrolled in this study were classified into three groups: Borrmann types I+II, III, and IV. The clinicopathological characteristics and prognosis were subsequently compared between Borrmann type IV and other types of GC.

### Classification of advanced gastric cancer according to Borrmann type

In consideration of the great heterogeneity of Borrmann classification by different endoscopic physicians during preoperative endoscopy in our institution, the definition of Borrmann classification was mainly based on intraoperative and postoperative macroscopic pathological assessments: type 1 (polypoid tumors with sharp demarcation from the adjacent mucosal tissue), type 2 (ulcerated carcinomas, with margins being sharp demarcation and raise), type 3 (ulcerated carcinomas with indefinite margins and infiltration into the surrounding wall), and type 4 (carcinomas with diffuse infiltration, where ulceration generally is not a characteristic) [13].

### Clinicopathological data

Data on patient demographics (gender and age), clinicopathological features (tumor size, tumor location, macroscopic type, tumor-node-metastasis (TNM) stage, differentiation, lymphovascular invasion, and perineural invasion), and treatment strategies (surgical types, combined organ resection, and postoperative chemotherapy) were collected from the database of the Department of Gastrointestinal Surgery of West China Hospital, Sichuan University, followed by analysis and comparison among GC patients with different Borrmann types. The TNM stage was evaluated in accordance with the eighth edition of the AJCC TNM classification [14].

### Surgical treatment

The principle of gastrectomy and lymphadenectomy was in line with the Japanese Classification of Gastric Carcinoma 3rd and 4th English edition by JGCA [13, 15]. D2/D2+ lymphadenectomy was routinely conducted in patients with advanced GC. Billroth I, Billroth II, and Roux-en-Y anastomoses were adopted for reconstruction in this study. Frozen pathological examination of the resection margin was also routinely performed during the operation. In the case of resection margin involvement in frozen assessment, additional resections would be performed if necessary. For potential curative resection, combined organ resection was selectively conducted. Moreover, postoperative chemotherapy was conducted based on TNM stage, patient’s willingness, and physical condition.

### Follow-up

The postoperative follow-up was mainly achieved by regular out-patient visits, e-mails, or telephone interviews. Follow-up information was updated until January 1, 2019. To be specific, patients were followed up every 3–6 months during the first 2 years, subsequently every 6–12 months during the next 3–5 years, and finally, annually. The follow-up included physical examination, tumor marker examination, endoscopy, and abdominal CT scanning. Patients lost to follow-up were due to the fact that patients changed their telephone number or refused reexamination in our hospital. Of the 2092 patients, 1872 (89.5%) were followed up.

### Statistical analysis

Student’s *t* test was utilized to analyze continuous data (shown as mean ± standard deviation). Categorical data were analyzed by the Fisher exact test or chi-square test. The Kaplan-Meier (KM) method was employed to plot survival curves, followed by comparison by a log-rank test. Both univariate and multivariate Cox’s proportional hazard regression models were performed. SPSS version 19.0 (IBM Corp., Armonk, NY, USA) was employed for statistical analysis. A two-sided *p* value < 0.05 suggested statistical significance.

## Results

Of the 2092 patients with advanced GC, 54(2.6%), 1042(49.8%), 850(40.6%) and 146 (7.0%) of them were assigned as Borrmann types I, II, III, and IV, respectively. The survival curves were not significantly different in GC patients between type I and type II (*p* = 0.712), or between type I and type III (*p* = 0.519) (Supplemental Figure). Afterwards, the clinicopathological characteristics were compared between type I and type II GC, and between type I and type III GC. Consequently, tumor size (*p* = 0.143), histologic type (*p* = 0.314), T stage (*p* = 0.243), N stage (*p* = 0.137), and TNM stage (*p* = 0.618) were not significantly different between type I and type II GC (Supplemental Table 1). However, tumor size (*p* = 0.009), N stage (*p* = 0.009), T stage (*p* < 0.001), and TNM stage (*p* < 0.001) were statistically significant between type I and type III GC (Supplemental Table 2). Those results suggested similar biological behaviors between Borrmann type I and type II GC. In consideration of the small sample of type I GC patients, patients with types I and II were combined, followed by comparison with type III and IV GC on clinicopathological features as well as survival.

Moreover, to exclude the bias due to different TNM stages in the three Borrmann groups, patients were further divided into four subgroups (TNM I, II, III, and IV), followed by a comparison of the overall survival (OS) among three Borrmann type patients in all subgroups after stratification. As a result, OS was not statistically significant among the three Borrmann groups in TNM stage I and stage II subgroup (*p* > 0.05). However, OS was significantly different among the three Borrmann groups in TNM stage III and IV subgroup (*p* < 0.05).

### Clinicopathological features

Tumor location, curative resection, histologic type, lymphovascular invasion, perineural invasion, and TNM stage were statistically significant in type III and IV GC in comparison with type I+II GC. Moreover, there were more patients with large tumor size in type III and IV GC (*p* < 0.001). In addition, type IV GC was more prevalent in female subjects (*p* < 0.001) and had a higher chance of involving the entire stomach (*p* < 0.001). Finally, type III and IV GC had more perineural invasion (*p* = 0.001), lymphovascular invasion (*p* < 0.05), undifferentiated histology (*p* < 0.05), noncurative resection, M stage, N stage, advanced T stage, and TNM stage (all *p* < 0.001) compared to those in type I+II GC (Table 1).

**Table 1:** Comparison of clinicopathological features among Borrmann type I+II, III, and IV tumors

Variables	Borrmann I+II, *N* = 1096 (%)	Borrmann III, *N* = 850 (%)	*p* value	Borrmann IV, *N* = 146 (%)	*p* value
Gender			0.761		< 0.001
Male	789 (72.0)	606 (71.3)		83 (56.8)	
Female	307 (28.0)	244 (28.7)		63 (43.2)	
Age, years			0.647		0.216
≤ 60	590 (53.8)	448 (52.7)		87 (59.6)	
> 60	506 (46.2)	402 (47.3)		59 (40.4)	
Tumor size, cm			< 0.001		< 0.001
≤ 5	715 (65.2)	314 (36.9)		17 (11.6)	
> 5	381 (34.8)	536 (63.1)		129 (88.4)	
Tumor location			< 0.001		< 0.001
Upper 1/3	359 (32.8)	254 (29.9)		38 (26.0)	
Middle 1/3	113 (10.3)	134 (15.8)		34 (23.3)	
Lower 1/3	619 (56.5)	439 (51.6)		27 (18.5)	
Entire	5 (0.5)	23 (2.7)		47 (32.2)	
Curative resection			< 0.001		< 0.001
R0	1032 (94.2)	726 (85.4)		93 (63.7)	
R1/2	64 (5.8)	124 (14.6)		53 (36.3)	
T stages			< 0.001		< 0.001
T2	283 (25.8)	76 (8.9)		5 (3.4)	
T3	289 (26.4)	166 (19.5)		10 (6.8)	
T4a	432 (39.4)	480 (56.5)		89 (61.0)	
T4b	82 (8.4)	128 (15.1)		42 (28.8)	
N stages			< 0.001		< 0.001
N0	296 (27.0)	123 (14.5)		7 (4.8)	
N1	207 (18.9)	130 (15.3)		12 (8.2)	
N2	218 (19.9)	190 (22.4)		13 (8.9)	
N3a	256 (23.4)	253 (29.8)		38 (26.0)	
N3b	119 (10.9)	154 (18.1)		76 (52.1)	
M stage			< 0.001		< 0.001
M0	1011 (92.2)	732 (86.1)		103 (70.5)	
M1	85 (7.8)	118 (13.9)		43 (29.5)	
TNM stages			< 0.001		< 0.001
I	133 (12.1)	21 (2.5)		0 (0.0)	
II	329 (30.0)	164 (19.3)		9 (6.2)	
III	549 (50.1)	547 (64.4)		94 (64.4)	
IV	85 (7.8)	118 (13.9)		43 (29.4)	
Histologic type			0.003		< 0.001
G1/G2	397 (36.2)	254 (29.9)		20 (13.7)	
G3/G4	699 (63.8)	596 (70.1)		126 (86.3)	
Lymphovascular invasion			0.044		< 0.001
Positive	172 (15.7)	163 (19.2)		47 (32.2)	
Negative	924 (84.3)	687 (80.8)		99 (67.8)	
Perineural invasion			0.001		0.001
Positive	138 (12.6)	151 (17.8)		34 (23.3)	
Negative	958 (87.4)	699 (82.2)		112 (76.7)	
Combined organ resection					0.556
Yes	55 (5.0)	44 (5.2)	0.917	9 (6.2)	
No	1041 (95.0)	806 (94.8)		137 (93.8)	
Postoperative chemotherapy			0.466		0.384
Yes	477 (43.5)	384 (45.2)		58 (39.7)	
No	619 (56.5)	466 (54.8)		88 (60.3)	

*G1/G2* well or moderately differentiated, *G3/G4* poorly or undifferentiated

### Survival analysis

**Fig. 2:**
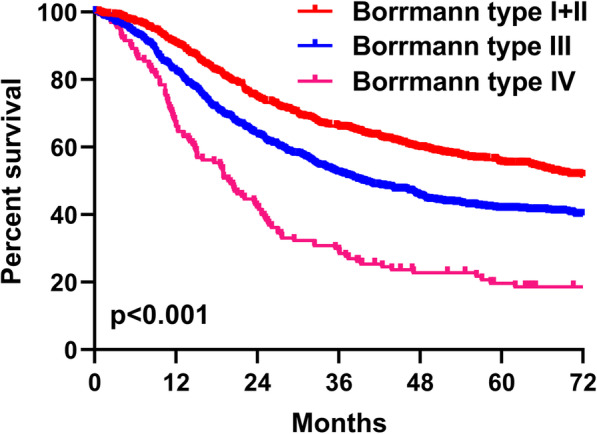
Comparation of survival curves between Borrmann type I+II, III, and IV gastric cancer

**Fig. 3:**
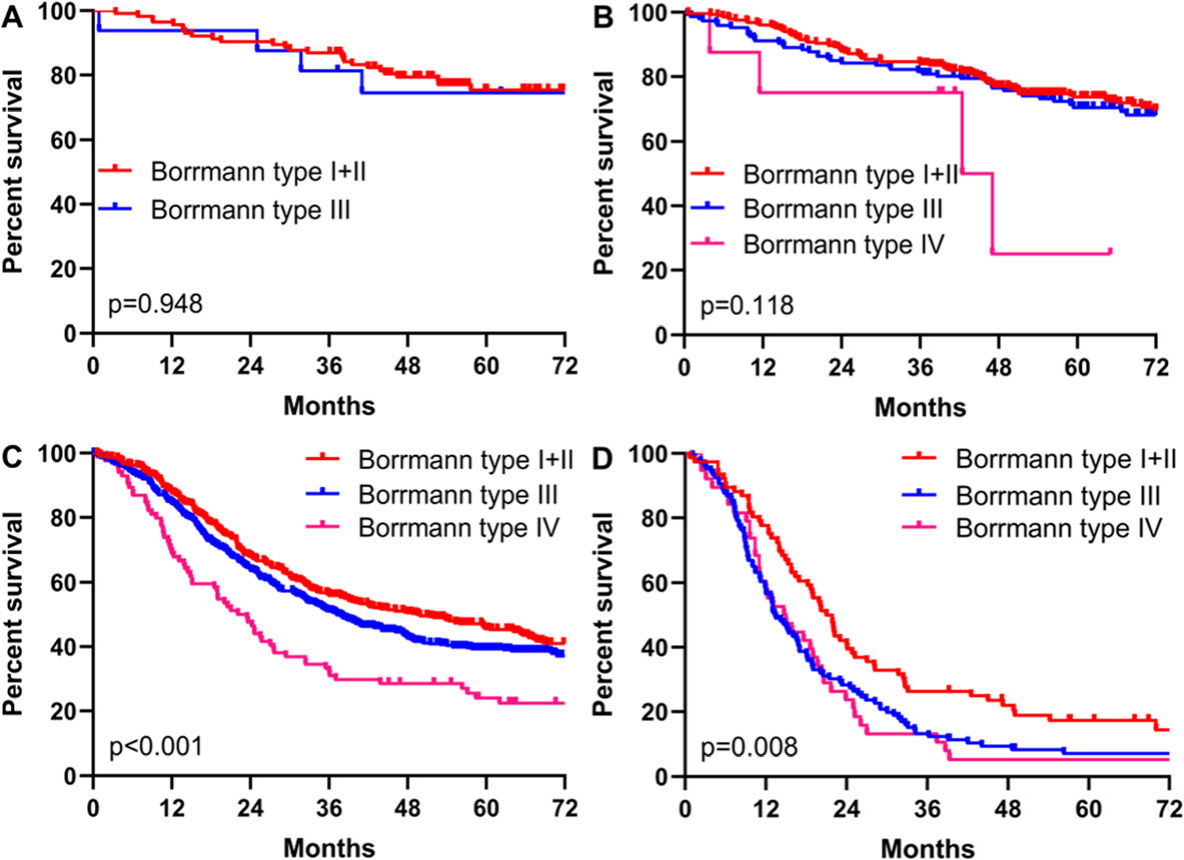
Comparation of survival curves between Borrmann type I+II, III, and IV gastric cancer in TNM stage I (**a**), II (**b**), III (**c**), and IV(**d**)

The 3-year and 5-year survival rates were 67.8% and 57.2% for type I+II GC, 59.0% and 48.5% for type III GC, and 37.9% and 28.9% for type IV GC, respectively, with significantly different survival curves (*p* < 0.001) (Fig. 2). In stratification analysis based on TNM stage, OS was significantly different among type I+II, type III, and type IV GC in TNM stage III (*p* < 0.001) (Fig. 3c) as well as stage IV (*p* = 0.008) (Fig. 3d), but not in stage II (*p* = 0.118) (Fig. 3b). Moreover, OS was insignificant between type I+II and type III tumors in TNM stage I (*p* = 0.948) (Fig. 3a).

**Fig. 4:**
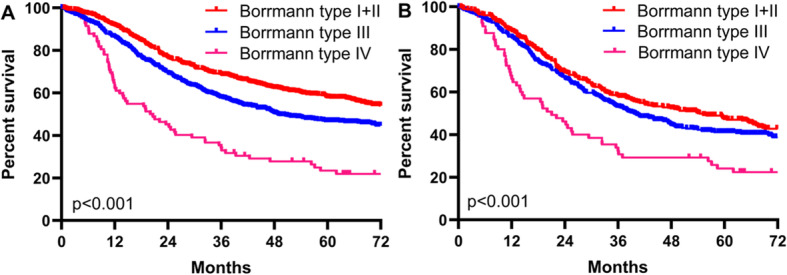
Comparation of survival curves between Borrmann type I+II, III, and IV gastric cancer in patients with curative resection (**a**) and in subgroup TNM III (**b**)

After curative resection, the 3-year and 5-year survival rates were 69.7% and 59.3% for type I+II GC, 62.1% and 51.6% for type III GC, and 46.7% and 35.7% for type IV GC, respectively, with significantly different survival curves among three groups (*p* < 0.001) (Fig. 4a). Stratification analysis according to the TNM stage revealed significantly different OS among type I+II, type III, and type IV GC only in TNM stage III (*p* < 0.001) (Fig. 4b).

**Fig. 5:**
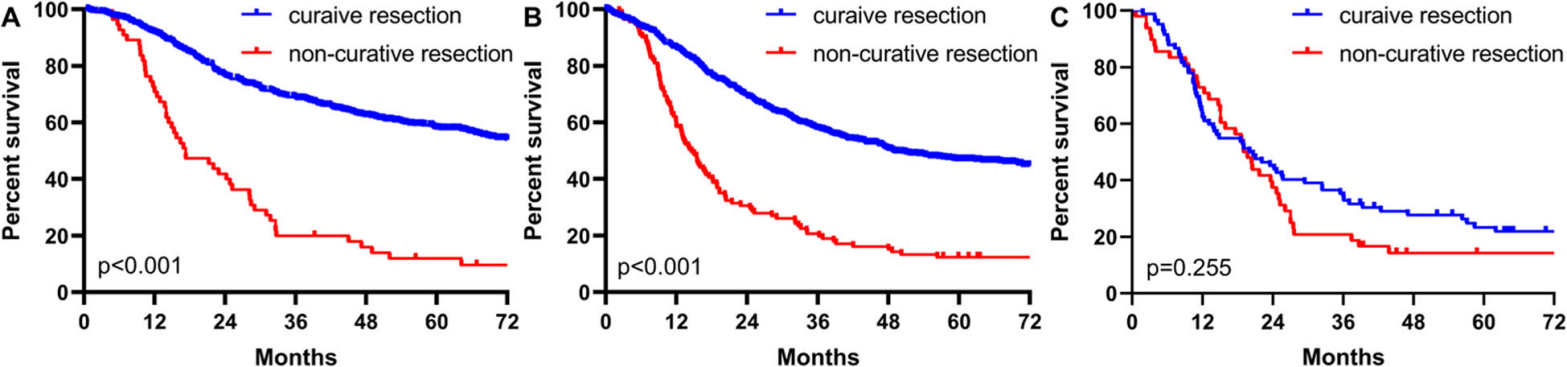
Comparation of survival curves between patients with curative resection and with noncurative resection in Borrmann I+II (**a**), Borrmann III (**b**), and Borrmann IV (**c**)

**Fig. 6:**
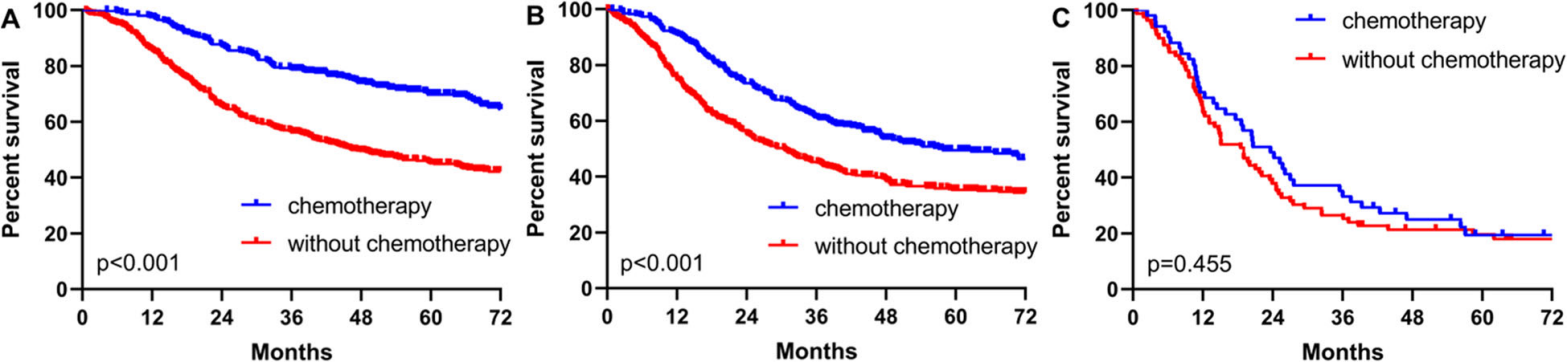
Comparation of survival curves between patients with chemotherapy and without chemotherapy in Borrmann I+II (**a**), Borrmann III (**b**), and Borrmann IV (**c**)

In addition, the OS rates of patients with types I+II and III undergoing curative resection were significantly higher than those receiving noncurative resection (*p* < 0.001) (Fig. 5a, b); however, the difference was insignificant in patients with type IV (*p* = 0.255) (Fig. 5c). The OS rates of patients with type I+II and type III GC receiving postoperative chemotherapy were significantly higher than those without postoperative chemotherapy (*p* < 0.001) (Fig. 6a, b), which was not significant in patients with type IV (Fig. 6c) (*p* = 0.455).

### Prognostic factors

For all patients, the univariate analysis indicated that histologic type; curative resection; Borrmann type; tumor size; lymphovascular invasion; postoperative chemotherapy; T, N, and M stages; and TNM stage (all *p* < 0.001) were closely related to OS in GC patients. Moreover, the multivariate Cox regression model revealed that Borrmann type (*p* = 0.01), postoperative chemotherapy (*p* < 0.001), curative resection (*p* = 0.018), and TNM stage (*p* < 0.001) were all independent predictors of OS among GC patients (Table 2).

**Table 2:** Prognostic factors of all patients with gastric cancer according to Cox proportional hazard analysis

Prognostic factors	Univariate		Multivariate	
	HR (95% CI)	*p* value	HR (95% CI)	*p* value
Borrmann type	1.389 (1.264–1.528)	< 0.001	1.138 (1.032–1.255)	0.010
Gender	1.021 (0.895–1.166)	0.755		
Age	1.085 (0.961–1.225)	0.189		
Tumor size	1.683 (1.485–1.906)	< 0.001	–	–
Tumor location	1.006 (0.943–1.073)	0.857		
Histologic type	1.288 (1.123–1.477)	< 0.001	–	–
Curative resection	2.160 (1.849–2.524)	< 0.001	1.240 (1.038–1.482)	0.018
T stages	1.573 (1.463–1.690)	< 0.001		
N stages	1.429 (1.362–1.498)	< 0.001		
M stages	2.214 (1.900–2.580)	< 0.001		
TNM stages	1.938 (1.773–2.118)	< 0.001	1.721 (1.555–1.904)	< 0.001
Lymphovascular invasion	1.418 (1.225–1.640)	< 0.001	–	–
Nerve invasion	1.149 (0.972–1.357)	0.104		
Postoperative chemotherapy	0.615 (0.540–0.699)	< 0.001	0.685 (0.602–0.780)	< 0.001

*HR* hazard ratio, *CI* confidence interval

We conducted a subgroup analysis on patients who undergone curative resection and with TNM stage III cancer. Univariate analyses showed that Borrmann type (*p* < 0.001), tumor size (*p* = 0.023), T stages (*p* < 0.001), N stages (*p* < 0.001), and postoperative chemotherapy (*p* < 0.001) were significantly related to the survival outcomes. In multivariate analyses, the results revealed that Borrmann type (*p* = 0.045), T stages (*p* < 0.001), N stages (*p* < 0.001), and postoperative chemotherapy (*p* < 0.001) were all independent predictors of OS among GC patients (Table 3).

**Table 3:** Prognostic factors of all patients in TNM stage III undergoing curative resection according to Cox proportional hazard analysis

Prognostic factors	Univariate		Multivariate	
	HR (95% CI)	*p* value	HR (95% CI)	*p* value
Borrmann type	1.636 (1.258–2.129)	< 0.001	1.319 (1.006–1.729)	0.045
Gender	0.980 (0.824–1.165)	0.816		
Age	1.101 (0.939–1.293)	0.236		
Tumor size	1.210 (1.027–1.426)	0.023	–	–
Tumor location	0.899 (0.778–1.038)	0.147		
Histologic type	1.084 (0.901–1.304)	0.391		
T stages	1.655 (1.342–2.040)	< 0.001	1.599 (1.292–1.978)	< 0.001
N stages	1.637 (1.384–1.936)	< 0.001	1.624 (1.369–1.926)	< 0.001
Lymphovascular invasion	1.186 (0.976–1.441)	0.086		
Nerve invasion	0.983 (0.791–1.223)	0.879		
Postoperative chemotherapy	0.633 (0.533–0.751)	< 0.001	0.638 (0.537–0.757)	< 0.001

*HR* hazard ratio, *CI* confidence interval

## Discussion

The Borrmann system (types I–IV) is widely adopted as the macroscopic classification of advanced GC. To be specific, the margins of both Borrmann type I and II GC have sharp demarcation, and Borrmann type III GC has indefinite limits [13]. Our present findings revealed no statistical significance of long-term survival between type I and type II, or between type I and type III. Further analysis indicated similar clinicopathological characteristics between type I and type II GC, such as tumor size, TNM stage, invasion depth, lymph node involvement, and distant metastasis, which was significantly different between type I and type III, consistent with the study by Li et al. [12]. The results indicated similar biological behaviors between type I and type II GC, which was different from type III GC. Thus, Borrmann type I and type II GC were combined, followed by a comparison with type III and IV GC in our research.

In the research by Li et al., the presence of type I+II, type III, and type IV in advanced GC was 28.1%, 58.9%, and 13.0%, respectively [12], which was 21.8%, 68.3%, and 8.32%, respectively, in the study by Huang et al. [11]. In the present research, the proportion of type I+II, III, and IV cases in advanced GC was 52.4%, 40.6%, and 7.0%, respectively, which was different from their study. It might be due to the discrepancy of clinicopathological features of GC in different regions and different populations of Borrmann types included in those studies. Previous reports demonstrated that type III and type IV GC had distinct clinicopathological characteristics, such as delayed diagnosis at the advanced stage, peritoneal seeding, massive lymph node involvement, and low rate of curative resection [9, 10, 12]. In our research, type III and type IV GC had larger tumor size, poor differentiation, more lymphovascular and perineural invasion, more noncurative resection, and more advanced TNM stage than type I and II GC, consistent with previous findings. Those clinicopathological features indicated that Borrmann type III and type IV GC were associated with cancer aggressiveness.

Certain researches showed Borrmann type as an independent prognostic indicator in advanced GC patients, as indicated by multivariate analysis [9, 11, 12], which was not validated by other studies [16, 17]. Herein, in our study, Borrmann type remained as an independent prognostic indicator in all GC patients, which was not an independent prognostic indicator for GC patients after curative resection. In our study, the noncurative resection rate in type I+II, III, and IV GC was 5.8%, 14.6%, and 36.3%, respectively. The relatively high proportion of noncurative resection in type III and IV tumor probably had certain effects on the prognostic significance of Borrmann type. Moreover, patients with type III and type IV GC had a worse prognosis than type I+II tumors, regardless of the curative or noncurative resection, consistent with previous outcomes [9, 12], which might be caused by more advanced TNM stage GC cases in type III and type IV. To control the confounding factor of noncurative resection, stratification analysis was used for GC patients with curative resection, suggesting a significant prognosis among the three Borrmann groups only in TNM stage III. Then, we conducted a subgroup analysis on patients undergoing curative resection and TNM stage III cancer. Our results revealed that macroscopic type was an independent prognostic factor in TNM stage III cancer. It was reported that compared with other types of gastric cancer, Borrmann IV GC had more lymph node metastasis and more frequent peritoneal recurrence after radical resection, suggesting more aggressive biologic behaviors, which may be the reasons for its poor prognosis [9–12]. Therefore, Borrmann type IV GC should be considered as a special subgroup of advanced gastric cancer. Clinicians should pay more attention to patients with type IV GC, especially those with TNM stage III, and the establishment of effective treatment for this population is necessary.

Curative resection is widely accepted as a critical prognostic indicator in advanced GC patients [12, 18, 19]. In this research, the multivariate analysis also demonstrated that curative resection was an independent prognostic factor. Among patients with type I+II and type III GC, the prognosis was better in those receiving curative resections than those who did not. However, in patients with type IV GC, curative resections could not significantly improve their survival, which was similar to the report by Kim et al. His study showed limited effects of surgery on Borrmann type IV GC because of the difficulty of performing curative resection, high peritoneal recurrence, and extremely poor tumor biology [20]. However, other studies reported that curative resection could improve the prognosis in patients with type IV GC, which was different from our study [12, 19]. It should be noted that the proportion of TNM stage was different in patients with type IV GC, and most patients with type IV GC had TNM III and IV stage; only 7.4% of patients with type IV GC had TNM stage II in our study. For patients with type IV at an early stage, curative surgery might prolong their survival; however, for those at an advanced stage, curative surgery might have limited prognostic significance. It was reported that early diagnosis of Borrmann type IV GC is essential for improving its prognosis [9–11]. For most cases of Borrmann type IV GC, there is no definite mass, so accurate preoperative endoscopic and CT examination are particularly important. Therefore, efforts should be made to detect lesions at an early stage, and curative resection should be performed for those patients. Up to date, the prognostic significance of curative gastrectomy for Borrmann type IV GC is still under debate, which should be further investigated.

Adjuvant chemotherapy could prolong the survival of patients with advanced GC [3, 21]. In our research, the multivariate survival analysis revealed that adjuvant chemotherapy was an independent prognostic indicator. However, different from patients with type I+II and type III GC, patients with type IV GC who received postoperative chemotherapy did not have significantly better survival rates than those with surgical resection alone, indicating that Borrmann type IV GC has special biological behavior. Several reports demonstrated low response rates for chemotherapy in patients with type IV GC [22, 23]. Some authors reported that Borrrmann type IV GC had a higher rate of signet ring cell carcinoma and showed less sensitivity to chemotherapy, which was similar with our result [24, 25]. This might be one of the reasons that the survival of patients with type IV GC could not be improved by adjuvant chemotherapy, which should be enhanced by the development of multimodality treatment [26, 27].

This study had some limitations: Firstly, in this retrospective study, possible selection bias and performance of analysis bias were unavoidable. Secondly, the number of Borrmann type I and IV GC was relatively limited from a single institute. Thus, a large-scale, well-designed prospective study should be performed to provide stronger evidence in this aspect.

## Conclusions

The clinicopathological features and prognosis of Borrmann type I+II, type III, and type IV GC were different. Therefore, Borrmann type can be simply employed as a valuable indicator to predict survival in advanced GC patients. More attention should be paid to the therapeutic strategies in type IV GC patients.

## Supplementary information

**Additional file 1.** Comparation of survival curves between Borrmann Type I, II and III gastric cancer (I vs II p=0.7122; I vs III p=0.5191; II vs III p<0.0001)

**Additional file 2: Table S1.** Comparison of clinicopathological features between Borrmann type I and II tumor in this study.

**Additional file 3: Table S2.** Comparison of clinicopathological features between Borrmann type I and III tumor in this study.

**Additional file 4: Table S3.** Comparison of clinicopathological features between Borrmann type III and IV tumor in this study.

**Additional file 5: Table S4.** Prognostic factors of gastric cancer patients with curative resection according to cox proportional hazard analysis.

## Data Availability

The data that support the results of this research is available on request from the corresponding author. Considering privacy or ethical restrictions, the data is not publicly available.

## Abbreviations

G1: Well differentiated
G2: Moderate differentiated
G3: Lower differentiated
G4: Undifferentiated
HR: Hazard ratio
CI: Confidence interval
GC: Gastric cancer
OS: Overall survival
TNM: Tumor-node-metastasis
