# Supplementary figures and images for: Prognostic impact of Borrmann classification on advanced gastric cancer: a retrospective cohort from a single institution in western China
Source: World J Surg Oncol. 2020 Aug 13;18:204. doi: 10.1186/s12957-020-01987-5 (PMC7427284; doi:10.1186/s12957-020-01987-5)

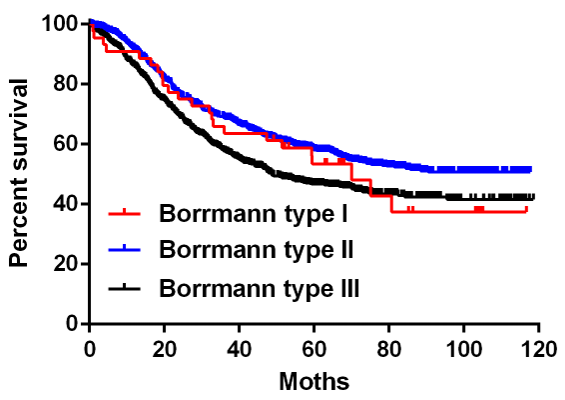

Supplement: Supplementary file 1 — Additional file 1. Comparation of survival curves between Borrmann Type I, II and III gastric cancer (I vs II p=0.7122; I vs III p=0.5191; II vs III p<0.0001) [file 12957_2020_1987_MOESM1_ESM.tif]
