# Supplementary material for: Prognostic impact of Borrmann classification on advanced gastric cancer: a retrospective cohort from a single institution in western China
Source: World J Surg Oncol. 2020 Aug 13;18:204. doi: 10.1186/s12957-020-01987-5 (PMC7427284; doi:10.1186/s12957-020-01987-5)
Supplement: Supplementary file 2 — Additional file 2: Table S1. Comparison of clinicopathological features between Borrmann type I and II tumor in this study. [file 12957_2020_1987_MOESM2_ESM.docx]

| **Supplement Table 1: Comparison of clinicopathological features between Borrmann type I and II tumor in this study** | | | |
| --- | --- | --- | --- |
| **Clinicopathological features** | **Borrmann type I group**  **N=54 (%)** | **Borrmann type II group**  **N=1042 (%)** | ***P* value** |
| Gender |  |  | 0.042 |
| Male | 32 (59.3) | 757 (72.6) |  |
| Female | 22 (40.7) | 285 (27.4) |  |
| Age, year |  |  | 0.675 |
| ≤60 | 31 (57.4) | 559 (53.6) |  |
| >60 | 23 (42.6) | 483 (46.4) |  |
| Tumor size, cm |  |  | 0.143 |
| ≤5 | 30 (55.6) | 685 (65.7) |  |
| >5 | 24 (44.4) | 357 (34.3) |  |
| Tumor location |  |  | 0.350 |
| Upper 1/3 | 20 (37.0) | 38 (32.5) |  |
| Middle 1/3 | 4 (7.4) | 34 (10.5) |  |
| Lower 1/3 | 29 (53.7) | 27 (56.6) |  |
| Entire | 1 (1.9) | 47 (0.4) |  |
| Curative resection |  |  |  |
| R0 | 50 (92.6) | 982 (94.2) | 0.550 |
| R1/2 | 4 (7.4) | 60 (5.8) |  |
| T stages |  |  | 0.243 |
| T2 | 19 (35.2) | 264 (25.3) |  |
| T3 | 16 (29.6) | 273 (26.2) |  |
| T4a | 15 (27.8) | 417 (40.0) |  |
| T4b | 4 (7.4) | 88 (8.4) |  |
| N stages |  |  | 0.137 |
| N0 | 13 (24.1) | 283 (27.2) |  |
| N1 | 11 (20.4) | 196 (18.8) |  |
| N2 | 17 (31.5) | 201 (19.3) |  |
| N3a | 11 (20.4) | 245 (23.5) |  |
| N3b | 2 (3.7) | 117 (11.2) |  |
| M stage |  |  | 0.672 |
| M0 | 49 (90.7) | 962 (92.3) |  |
| M1 | 5 (9.3) | 80 (7.7) |  |
| TNM stages |  |  | 0.618 |
| I | 6 (11.1) | 127 (12.2) |  |
| II | 20 (37.0) | 309 (29.7) |  |
| III | 23 (42.6) | 526 (50.5) |  |
| IV | 5 (9.3) | 80 (7.7) |  |
| Histologic type |  |  | 0.314 |
| G1/G2 | 23 (42.6) | 374 (35.9) |  |
| G3/G4 | 31 (57.4) | 668 (64.1) |  |
| Lymphovascular invasion |  |  | 0.572 |
| Positive | 47 (87.0) | 877 (84.2) |  |
| Negative | 7 (13.0) | 165 (15.8) |  |
| Perineural invasion |  |  | 0.993 |
| Positive | 47 (78.0) | 911 (87.4) |  |
| Negative | 7 (13.0) | 131 (12.6) |  |
| Combined organ resection |  |  |  |
| Yes | 99 (5.1) | 9 (6.2) | 0.559 |
| No | 1847 (94.9) | 137 (93.8) |  |
| Postoperative chemotherapy |  |  | 0.888 |
| Yes | 31 (57.4) | 588 (56.4) |  |
| No | 23 (42.6) | 454 (43.6) |  |
| Abbreviations: G1/G2: well or moderately differentiated; G3/G4: poorly or undifferentiated | | | |
|  | | | |
