# Supplementary material for: Prognostic impact of Borrmann classification on advanced gastric cancer: a retrospective cohort from a single institution in western China
Source: World J Surg Oncol. 2020 Aug 13;18:204. doi: 10.1186/s12957-020-01987-5 (PMC7427284; doi:10.1186/s12957-020-01987-5)
Supplement: Supplementary file 3 — Additional file 3: Table S2. Comparison of clinicopathological features between Borrmann type I and III tumor in this study. [file 12957_2020_1987_MOESM3_ESM.docx]

| **Supplement Table 2: Comparison of clinicopathological features between Borrmann type I and III tumor in this study** | | | |
| --- | --- | --- | --- |
| **Clinicopathological features** | **Borrmann type I group**  **N=54 (%)** | **Borrmann type III group**  **N=850 (%)** | ***P* value** |
| Gender |  |  | 0.065 |
| Male | 32 (59.3) | 606 (71.3) |  |
| Female | 22 (40.7) | 244 (28.7) |  |
| Age, year |  |  | 0.502 |
| ≤60 | 31 (57.4) | 448 (52.7) |  |
| >60 | 23 (42.6) | 402 (47.3) |  |
| Tumor size, cm |  |  | 0.009 |
| ≤5 | 30 (55.6) | 314 (36.9) |  |
| >5 | 24 (44.4) | 536 (63.1) |  |
| Tumor location |  |  | 0.339 |
| Upper 1/3 | 20 (37.0) | 254 (29.9) |  |
| Middle 1/3 | 4 (7.4) | 134 (15.8) |  |
| Lower 1/3 | 29 (53.7) | 439 (51.6) |  |
| Entire | 1 (1.9) | 23 (2.7) |  |
| Curative resection |  |  |  |
| R0 | 50 (92.6) | 726 (85.4) | 0.163 |
| R1/2 | 4 (7.4) | 124 (14.6) |  |
| T stages |  |  | <0.001 |
| T2 | 19 (35.2) | 76 (8.9) |  |
| T3 | 16 (29.6) | 166 (19.5) |  |
| T4a | 15 (27.8) | 480 (56.5) |  |
| T4b | 4 (7.4) | 128 (15.1) |  |
| N stages |  |  | 0.009 |
| N0 | 13 (24.1) | 123 (14.5) |  |
| N1 | 11 (20.4) | 130 (15.3) |  |
| N2 | 17 (31.5) | 190 (22.4) |  |
| N3a | 11 (20.4) | 253 (29.8) |  |
| N3b | 2 (3.7) | 154 (18.1) |  |
| M stage |  |  | 0.337 |
| M0 | 49 (90.7) | 732 (86.1) |  |
| M1 | 5 (9.3) | 118 (13.1) |  |
| TNM stages |  |  | <0.001 |
| I | 6 (11.1) | 21 (2.5) |  |
| II | 20 (37.0) | 164 (19.3) |  |
| III | 23 (42.6) | 547 (64.4) |  |
| IV | 5 (9.3) | 118 (13.9) |  |
| Histologic type |  |  | 0.067 |
| G1/G2 | 23 (42.6) | 254 (29.9) |  |
| G3/G4 | 31 (57.4) | 596 (70.1) |  |
| Lymphovascular invasion |  |  | 0.368 |
| Positive | 47 (87.0) | 687 (80.8) |  |
| Negative | 7 (13.0) | 163 (19.2) |  |
| Perineural invasion |  |  | 0.368 |
| Positive | 47 (87.0) | 699 (82.2) |  |
| Negative | 7 (13.0) | 151 (17.8) |  |
| Combined organ resection |  |  |  |
| Yes | 99 (5.1) | 9 (6.2) | 0.559 |
| No | 1847 (94.9) | 137 (93.8) |  |
| Postoperative chemotherapy |  |  | 0.779 |
| Yes | 23 (42.6) | 384 (45.2) |  |
| No | 31 (57.4) | 466 (54.8) |  |
| Abbreviations: G1/G2: well or moderately differentiated; G3/G4: poorly or undifferentiated | | | |
|  | | | |
