# Supplementary material for: Prognostic impact of Borrmann classification on advanced gastric cancer: a retrospective cohort from a single institution in western China
Source: World J Surg Oncol. 2020 Aug 13;18:204. doi: 10.1186/s12957-020-01987-5 (PMC7427284; doi:10.1186/s12957-020-01987-5)
Supplement: Supplementary file 4 — Additional file 4: Table S3. Comparison of clinicopathological features between Borrmann type III and IV tumor in this study. [file 12957_2020_1987_MOESM4_ESM.docx]

| **Supplement Table 3: Comparison of clinicopathological features between Borrmann type III and IV tumor in this study** | | | |
| --- | --- | --- | --- |
| **Clinicopathological features** | **Borrmann type III group**  **N=850 (%)** | **Borrmann type IV group**  **N=146 (%)** | ***P* value** |
| Gender |  |  | <0.001 |
| Male | 606 (71.3) | 83 (56.8) |  |
| Female | 244 (28.7) | 63 (43.2) |  |
| Age, year |  |  | 0.123 |
| ≤60 | 448 (52.7) | 87 (59.6) |  |
| >60 | 402 (47.3) | 59 (40.4) |  |
| Tumor size, cm |  |  | <0.001 |
| ≤5 | 314 (36.9) | 17 (11.6) |  |
| >5 | 536 (63.1) | 129 (88.4) |  |
| Tumor location |  |  | <0.001 |
| Upper 1/3 | 254 (29.9) | 38 (26.0) |  |
| Middle 1/3 | 134 (15.8) | 34 (23.3) |  |
| Lower 1/3 | 439 (51.6) | 27 (18.5) |  |
| Entire | 23 (2.7) | 47 (32.2) |  |
| Curative resection |  |  | <0.001 |
| R0 | 726 (85.4) | 93 (63.7) |  |
| R1/2 | 124 (14.6) | 53 (36.3) |  |
| T stages |  |  | <0.001 |
| T2 | 76 (8.9) | 5 (3.4) |  |
| T3 | 166 (19.5) | 10 (6.8) |  |
| T4a | 480 (56.5) | 89 (61.0) |  |
| T4b | 128 (15.1) | 42 (28.8) |  |
| N stages |  |  | <0.001 |
| N0 | 123 (14.5) | 7 (4.8) |  |
| N1 | 130 (15.3) | 12 (8.2) |  |
| N2 | 190 (22.4) | 13 (8.9) |  |
| N3a | 253 (29.8) | 38 (26.0) |  |
| N3b | 154 (18.1) | 76 (52.1) |  |
| M stage |  |  | <0.001 |
| M0 | 732 (86.1) | 103 (70.5) |  |
| M1 | 118 (13.9) | 43 (29.5) |  |
| TNM stages |  |  | <0.001 |
| I | 21 (2.5) | 0 (0) |  |
| II | 164 (19.3) | 9 (6.1) |  |
| III | 547 (64.4) | 94 (64.4) |  |
| IV | 118 (13.9) | 43 (29.5) |  |
| Histologic type |  |  | <0.001 |
| G1/G2 | 254 (29.9) | 20 (13.7) |  |
| G3/G4 | 596 (70.1) | 126 (86.3) |  |
| Lymphovascular invasion |  |  | <0.001 |
| Positive | 163 (19.2) | 47 (32.2) |  |
| Negative | 687 (80.8) | 99 (67.8) |  |
| Perineural invasion |  |  | 0.134 |
| Positive | 151 (17.8) | 34 (23.3) |  |
| Negative | 699 (82.2) | 112 (76.7) |  |
| Combined organ resection |  |  |  |
| Yes | 44 (5.2) | 9 (6.2) | 0.623 |
| No | 806 (94.8) | 137 (93.8) |  |
| Postoperative chemotherapy |  |  | 0.241 |
| Yes | 466 (54.8) | 58 (39.7) |  |
| No | 384 (45.2) | 88 (60.3) |  |
| Abbreviations: G1/G2: well or moderately differentiated; G3/G4: poorly or undifferentiated | | | |
|  | | | |
