# Supplementary material for: Prognostic impact of Borrmann classification on advanced gastric cancer: a retrospective cohort from a single institution in western China
Source: World J Surg Oncol. 2020 Aug 13;18:204. doi: 10.1186/s12957-020-01987-5 (PMC7427284; doi:10.1186/s12957-020-01987-5)
Supplement: Supplementary file 5 — Additional file 5: Table S4. Prognostic factors of gastric cancer patients with curative resection according to cox proportional hazard analysis. [file 12957_2020_1987_MOESM5_ESM.docx]

| **Table 4. Prognostic factors of gastric cancer patients with curative resection according to cox proportional hazard analysis** | | | | |
| --- | --- | --- | --- | --- |
|  | **Univariate** | | **Multivariate** | |
| **Prognostic factors** | **HR (95% CI)** | ***P* value** | **HR (95% CI)** | ***P* value** |
| Borrmann type | 1.318(1.181-1.471) | <0.001 | 1.081(0.962-1.215) | 0.190 |
| Gender | 0.986(0.850-1.143) | 0.848 |  |  |
| Age | 1.117(0.976-1.278) | 0.109 |  |  |
| Tumor size | 1.575(1.375-1.805) | <0.001 | 1.142(0.983-1.327) | 0.083 |
| Tumor Location | 0.983(0.914-1.056) | 0.636 |  |  |
| Histologic type | 1.279(1.101-1.485) | 0.001 | 1.059(0.908-1.234) | 0.466 |
| T stages | 1.535(1.417-1.663) | <0.001 |  |  |
| N stages | 1.439(1.365-1.516) | <0.001 |  |  |
| TNM stages | 1.930(1.735-2.148) | <0.001 | 1.727(1.539-1.938) | <0.001 |
| Lymphovascular invasion | 1.408(1.188-1.670) | <0.001 | 1.161(0.977-1.380) | 0.090 |
| Nerve invasion | 1.105(0.910-1.342) | 0.314 |  |  |
| Postoperative chemotherapy | 0.568(0.492-0.656) | <0.001 | 0.634(0.548-0.733) | <0.001 |
| Abbreviations: HR: hazard ratio; CI: confidence interval | | | | |
